# Reform in Tuberculosis Work

**Published:** 1917-01-13

**Authors:** 


### REFORM IN TUBERCULOSIS WORK.

### I. The Sanatorium.

By A TUBERCULOSIS WORKER.

If any subject is topical at the present time, it is attempt at reform, reconstruction, amelioration. As to whether the most conspicuous of such current efforts are well judged or not, we most naturally say not a word. For indeed we concede no priority of politics to science, holding rather the opposite opinion, and remembering the dictum of one of the most philosophic of tribunes that humanity has more to expect from the progress of science than from any arrangement reached by councils or assemblies. If the lyric poet can praise dreams and decry "dusty deeds," so in his different way may the medical scientist look beyond everyday turmoil to the fruitful region of the scientific imagination. Notwithstanding, however, much tactical advantage can be gained by taking, as it were, every man in his humour, and that that humour just now is a reforming humour cannot be disputed. It may chance that the campaign against disease may, when closely observed, reveal faults just as that other campaign deemed greater has.

The most serious disease known to mankind is tuberculosis. A preventive and curative anti-tuberculosis campaign has had a prominent place in medico-sociological work for some time now, and on the surface everything seems satisfactory. A central commission drew up a general scheme of operations to be put into practice by local authorities and their advisers all over the country. Something of the kind has duly taken place. In most large areas subscriptions have been collected, rates levied, inspections made, sub-committees chosen, buildings erected, medical officers appointed, favourable reports issued. The superficial observer (a description that must necessarily apply to most laymen) would say that, subject to the limitations of human knowledge, everything had been planned for the best, and, with the same reservation, that everything was going well. Now let us give straightaway some uncensored truth regarding those institutions which form an important unit in the anti-tuberculosis offensive—namely, public sanatoria—taking instances occurring within the last eight years at places distributed over most of the United Kingdom, and coming, unsought for, within the narrow circle of a single experience.

A was elected out of several competitors for a resident medical officership to the sanatorium of a consumption hospital. Two years later the post was advertised as being vacant. In reply to a letter, A gave every information within his power to the prospective applicant, adding, however, a remark that in any dispute committees always backed up a woman against a man. The inference drawn was that his leaving had something to do with a difference with the matron, a surmise that was confirmed later by a tuberculosis nurse who had been at the institution in question at the time of the events just related.

Some committee members of a sanatorium, visiting another institution, told the resident there that B, formerly a valued officer of their own, had relinquished the superintendentship of a third sanatorium against his will.

A year or two later, other visitors, a provincial medical consultant and a medical officer of health, similarly gave the information that C had been asked to resign from the senior post at their sanatorium. To a question as to whether this was due to any-thing discreditable on C's part the reply was at once in the negative. Patients had been grumbling about the food, and the committee thought the medical superintendent and the matron did not agree, so practically dismissed them both and the housekeeper in addition.

D was appointed medical superintendent to the sanatorium X. After two years he left to become an assistant to a medium-sized private sanatorium, at a small salary. At one period X had had six superintendents in four years.

Concerning another sanatorium, still better known for frequent changes (alleged to be due to the influence of an uneducated male official), the secretary of a consumption hospital, where E had formerly been a resident medical officer, stated that E told him in a letter that he had "got the sack." A physician of the same hospital opined that the fault was not E's. Subsequently E took office at X and soon became county tuberculosis officer.

A sanatorium residentship was advertised. In answer to a letter, F (formerly assistant to C) wrote that he had had differences with a new committee of management, and that his assistant was not a candidate for the post he was vacating.

A gentleman who was making a round of sanatorium visits asked the resident G how long he had been in office. The reply "Five years" evoked an expression of surprise, and a compliment as to the great amount of tact thus evinced. Some of the above instances, and one or two others, were within G's knowledge, so that the remark impressed him. He took occasion to leave before long. His predecessor's stay had been a year and a half; that of his first successor was six months, and of his second not quite two years.

Now the medical men who thus failed to establish connection possessed credentials which obviously passed the test of competition. All were physically well up to their work. Among them were three graduates of London, one of Edinburgh, one of Oxford. So far as is known, none had been guilty of any grave fault meriting dismissal; one perhaps had been absent from the institution pleasure-seeking more than was right. Moreover, the institutions concerned are not insignificant places, but, on the contrary, important and fairly well known in the tuberculosis world. It is thus on particular as well as on general grounds likely that medical men who take up sanatorium work are not the scum of the profession. But the state of frequent friction which these instances evince is well known to the initiated. Even in cases that might at first sight be used as arguments to the contrary, even in prominent cases of success, the hero, in Emersonian phrase, has at last become a bore. The only steady, harmonious tenure of a sanatorium residentship known to the writer—this is not to say that no others exist—is one which it is hoped to touch on in a future article.

